# Everything Is Prediction: Modern Machine Learning as Bayesian Inference

**DOI:** 10.3390/e28080869

**Published:** 2026-08-01

**Authors:** Nicholas G. Polson, Vadim Sokolov, Refik Soyer

**Affiliations:** 1 Booth School of Business, University of Chicago, Chicago, IL 60637, USA; ngp@chicagobooth.edu; 2 Department of Systems Engineering and Operations Research, George Mason University, Fairfax, VA 22030, USA; vsokolov@gmu.edu; 3 Department of Decision Sciences, School of Business, George Washington University, Washington, DC 20052, USA

**Keywords:** predictive inference, exchangeability, martingale posteriors, generative Bayesian computation, conformal prediction, in-context learning, diffusion models, proper scoring rules, Bregman divergence, maximum-entropy reinforcement learning, entropy, two cultures

## Abstract

We argue that the core methods of modern machine learning—conformal prediction, large language models and in-context learning, and generative/diffusion models—are not rivals to Bayesian inference but implementations of it, almost always of its *predictive* (de Finetti) form rather than its parameter-centric (prior-to-posterior) form. (1) Background: A quarter-century after Breiman contrasted the “data-modeling” and “algorithmic” cultures of statistics, we revisit that dichotomy and argue that the predictive view dissolves it—both cultures target the one-step-ahead density $p(y_{n+1}|y_{1:n})$, differing only in how they compute it. (2) Methods: We organize the modern toolkit around this predictive object and around *amortization*—replacing per-dataset inference with a single map learned by simulation—using Generative Bayesian Computation (GBC) as the connective spine. (3) Results: Conformal prediction, autoregressive language models, prior-data fitted networks, and score-based diffusion are each shown to construct, calibrate, or sample from the predictive object, summarized in a single “Rosetta” table; because all are fit by proper scoring rules—equivalently, by Bregman divergences—the information-theoretic frame is the natural unifier. (4) Conclusions: The equivalence is exact in idealized limits, asymptotic under exchangeability and its martingale relaxations, and measurably approximate for trained models—a three-grade taxonomy that we make explicit, row by row, and that bounds the thesis: prediction is not attribution, and the predictive view is deliberately silent about causal structures.

## 1. Introduction

The history of statistical learning is, in large part, an argument about what the primitive object of inference should be. Breiman [1] framed it as two cultures: a *data-modeling* culture that posits a stochastic mechanism and estimates its parameters, and an *algorithmic* culture that treats the mechanism as unknown and optimizes predictive accuracy directly. Efron [2] refined the distinction into a trichotomy—prediction, estimation, and attribution—and observed that modern algorithms excel at the first while remaining silent or unreliable on the third. A generation earlier, Wasserman [3,4] had argued that classical statistics and statistical machine learning are two dialects of a single language.

This paper advances the next-generation form of that claim: the algorithmic culture wins on prediction precisely when, and because, it is implicitly performing *amortized predictive Bayesian inference*. By “predictive”, we mean the de Finetti stance in which the one-step-ahead density $p(y_{n+1}|y_{1:n})$ is primitive and the parameter θ is a derived, asymptotic functional. By “amortized’,’ we mean that the cost of inference is paid once, up front, by training a single map—on simulated draws or on a large corpus—which is then evaluated by a forward pass on any new input, rather than re-run for each dataset. Where a classical Bayesian integrates a likelihood against a prior to obtain a posterior and then a predictive, the modern learner skips the middle step: it learns the predictive—or a transport map to the posterior—directly. Generative Bayesian Computation (GBC) [5] is the sharpest formulation of this move, and we use it as the connective spine.

Scope of the thesis.

The title is a slogan, and slogans overreach, so we state the claim in its careful form at the outset rather than qualifying it only in the discussion. What we argue is that *when a modern method succeeds at prediction, the object it has learned is a predictive distribution, and the calculus that governs it is Bayesian*. We do *not* argue that prediction exhausts the aims of statistics. Estimation, attribution, causal identification and scientific explanation are distinct inferential goals; they are not automatically delivered—and in some cases are actively obscured—by a well-calibrated predictive machine. A model that predicts an outcome from a covariate need not license an intervention on it, and no amount of held-out accuracy converts p(y∣x) into p(y∣do(x)). Throughout this paper, we therefore separate the predictive claim, which we defend, from the inferential-completeness claim, which we deny, and we return to the boundary in Section Where Prediction Is Not Enough and Section 12.

Contributions, and what is new relative to existing reviews.

Existing surveys—of Bayesian deep learning, of simulation-based inference [6], of conformal prediction [7], of variational methods [8]—are organized around a *method family*. Our organizing axis is instead the *target* ($p(y_{n+1}|y_{1:n})$) and the *computational bargain* (amortization). We contribute: (i) a reading of Breiman’s dichotomy on which the two cultures share a target and differ only in route (Section 2). (ii) a single dictionary—the Rosetta table of Section 10—pinning seven method families to four coordinates: the predictive object they represent, the proper score that trains them, where their uncertainty lives, and where their prior hides; (iii) an explicit three-grade taxonomy (Exact, Asymptotic, ≈approximate) attached to every row, so that the equivalences are auditable rather than rhetorical (Section 10.1); (iv) the identification of measure transport as the shared mechanism (Section 8) and of relative entropy—and its parent, the Bregman class (Section 9.1)—as the shared currency; and (v) a delineation of where the framework stops (Section Where Prediction Is Not Enough).

Our contribution is synthetic rather than technical. We revisit the two cultures (Section 2) and the predictive foundations that make the thesis precise (Section 3); describe the generative/amortized turn, with GBC at its center (Section 4); reread deep learning (Section 5), conformal prediction (Section 6), language models and in-context learning (Section 7), and diffusion (Section 7.3) as special cases; show the generative methods to be one family of measure transports (Section 8) with entropy as their common currency (Section 9); and assemble the predictive calculus (Section 10) before turning to its limits (Section 11 and Section 12).

**Figure 1:**
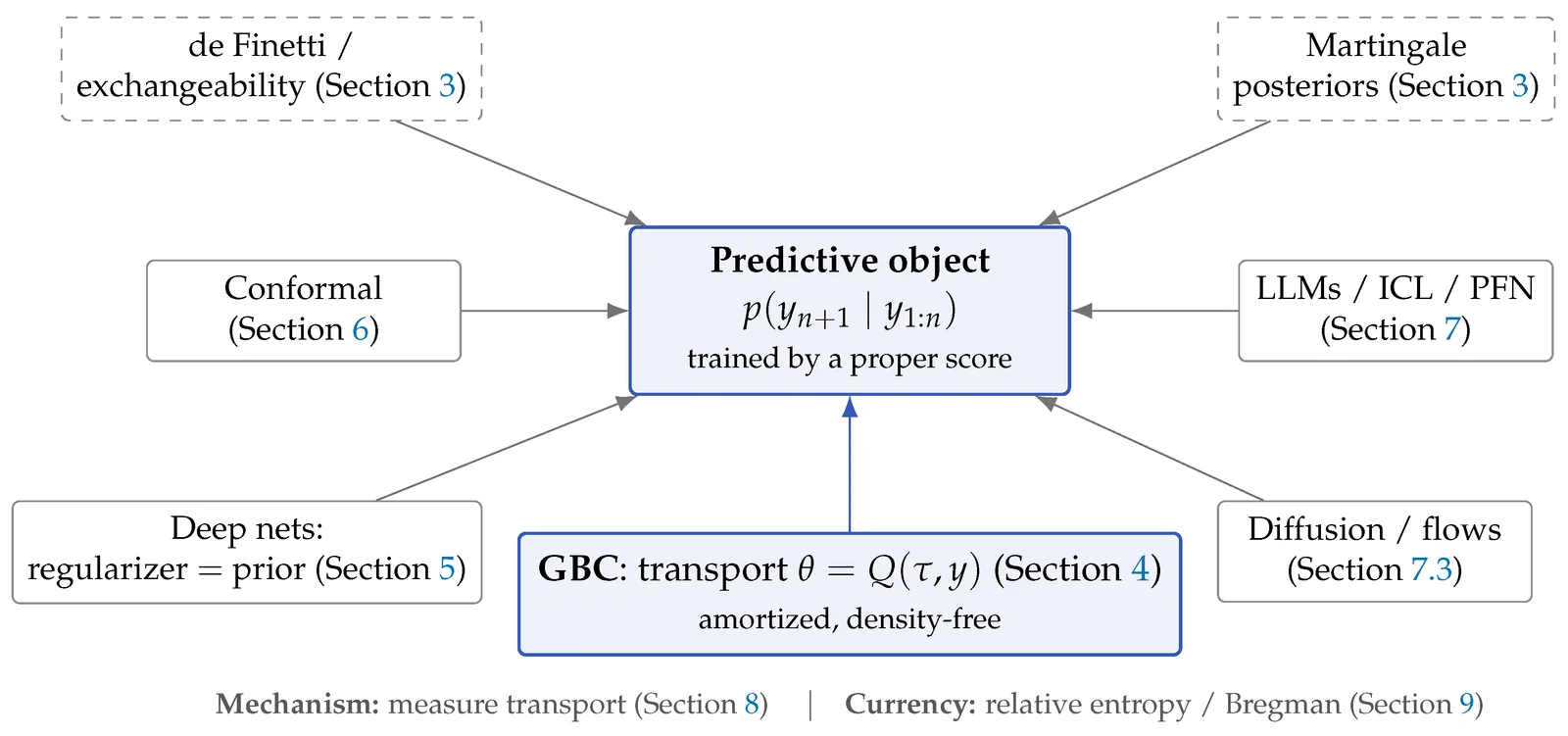
Road map. Every method in this survey constructs, calibrates, or samples the same predictive object; the foundations (dashed) say why that object is primitive, GBC supplies the computational spine, transport is the shared mechanism, and relative entropy is the shared currency. The Rosetta table of Section 10 is this figure in tabular form.

A single throughline connects the sections. Fix the predictive object $p(y_{n+1}|y_{1:n})$; ask, for each method, how it is *represented* (a sequence of conditionals, a transport map, a prediction set, a score), how it is *trained* (which proper scoring rule), and where its prior *hides* (a simulation distribution, a pretraining corpus, a regularizer, a base measure). The recurring answer is that the method amortizes—learns the map once, over a distribution of problems—and that the training objective, the regularizer, and the evaluation metric are all relative entropies. Those questions, applied method by method, are summarized in the Rosetta table of Section 10, and the road map of Figure 1 shows how the pieces fit together before we assemble them.

## 2. The Two Cultures, Revisited

Breiman [1] drew a now-famous line through the discipline. In the data-modeling culture, one assumes the data are generated by a stochastic model—linear regression, logistic regression, the Cox model—estimates its parameters, and validates by goodness-of-fit and significance tests. In the algorithmic culture, one treats the data mechanism as unknown, fits a flexible black box—random forests, boosting, neural networks—and judges it solely by predictive accuracy on held-out data. Breiman’s polemic was that the field had over-committed to the first culture and, by equating an estimated model with the truth, had paid for interpretability with accuracy and with conclusions that did not generalize. The published discussion—measured replies from Cox, Efron, Hoadley, and Parzen—conceded the predictive point while warning that prediction without mechanism abandons explanation and causal attribution.

The intervening quarter-century vindicated Breiman on accuracy and sharpened the conceptual map. Shmueli [9] separated explanatory from predictive modeling and showed that a model optimized to explain (minimum bias, correct mechanism) is generally not the model that predicts best (favorable bias–variance trade-off), so the two goals select different models even from the same data. Donoho [10], surveying “50 years of data science,” located the engine of the algorithmic culture’s success in the common-task framework of shared benchmarks and held-out scoring, which turns prediction into a measurable, competitive science. Vapnik [11] had already supplied that culture’s theory, in which generalization, not parameter recovery, is the object of study. Efron [2] closed the loop by adding attribution as a third aim and noting that pure prediction machines are weakest exactly there.

**Figure 2:**
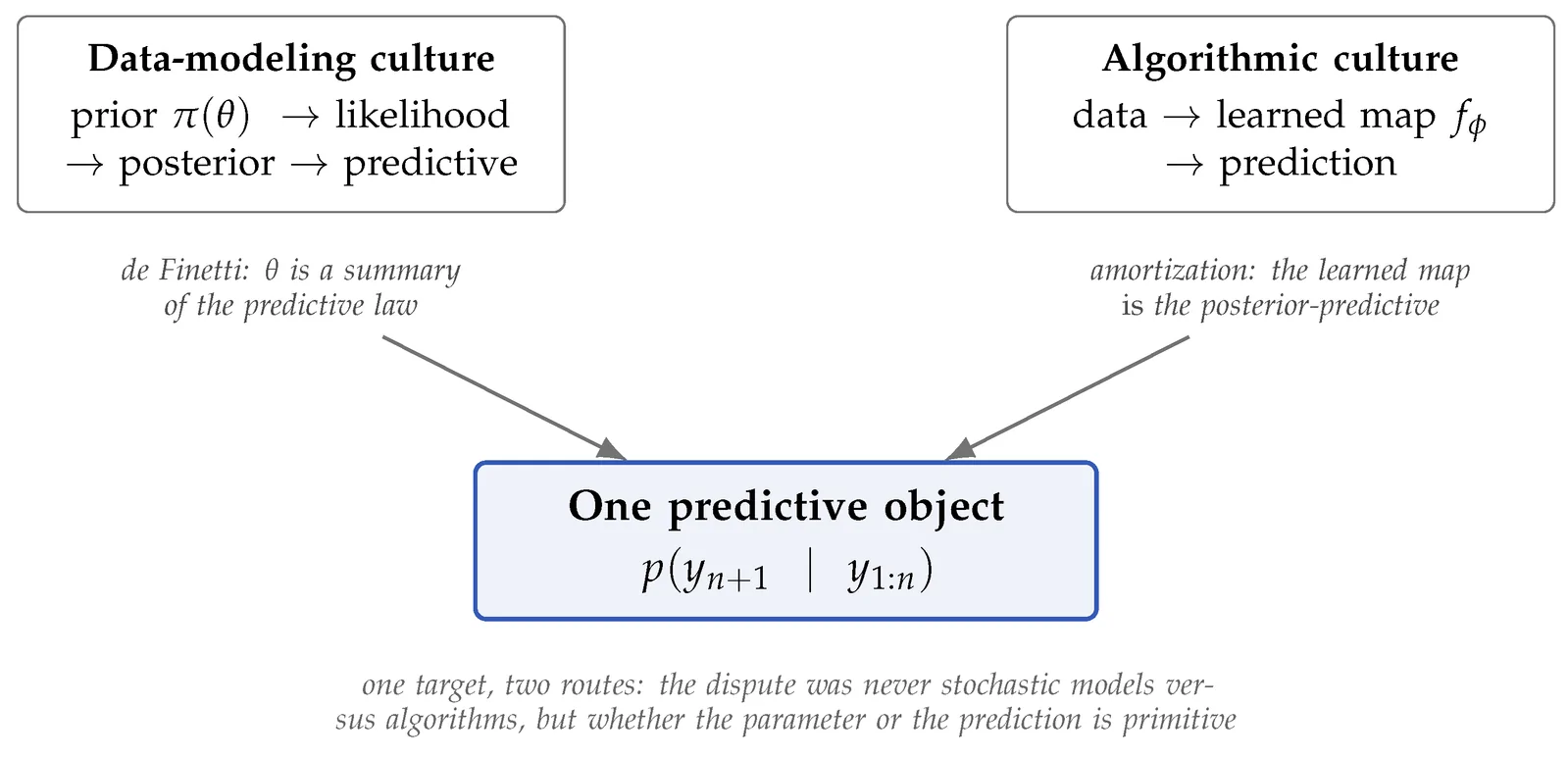
The two cultures of Breiman [1] collapse onto a single predictive target. The data-modeling route reaches $p(y_{n+1}|y_{1:n})$ by marginalizing a posterior; the algorithmic route reaches it by evaluating a learned map. De Finetti’s representation and amortization identify the two.

Our claim is that the dichotomy, real as a description of practice, dissolves at the level of targets. Both cultures produce a predictive distribution for the next observation and differ only in route. The data-modeling culture routes through the triple (prior, likelihood, posterior) and marginalizes to a predictive; the algorithmic culture trains a map from inputs to outputs and reads predictions off directly. De Finetti’s theorem (Section 3) shows the parameter θ of the first route to be only a compressed summary of an exchangeable predictive law—the “model” is a device for organizing predictions, not an end in itself. Amortization (Section 4 and Section 7) shows the learned map of the second route to be the posterior or posterior-predictive computed ahead of time, over a distribution of datasets, rather than rediscovered for each one. There is one predictive calculus, then, and two strategies for evaluating it (Figure 2). The genuine residue of the dichotomy is attribution: the predictive view is by construction silent about the identifiability and causal meaning of θ. This residue is not a footnote to our thesis but its boundary, and we treat it as such in Section Where Prediction Is Not Enough.

A concrete instance fixes intuition. Asked to predict a response from covariates, the data-modeling culture fits a linear or generalized linear model and reports coefficients with standard errors; the algorithmic culture fits a gradient-boosted ensemble or a neural network and reports held-out error. Both, however, are estimating the same conditional law p(y∣x), and the modern Bayesian object—a posterior predictive averaged over models or a single amortized map trained to emit it—contains both as limiting cases: linear-Gaussian structure where the data support it, flexible nonlinearity where they do not. The common-task framework that Donoho [10] credits for the algorithmic culture’s ascent is, on this reading, a tournament of predictive distributions scored by a proper rule—machine learning’s rediscovery of the prequential principle of Dawid [12].

The algorithmic culture even had a predictive-first theory from the outset. Vapnik [11] argued for transduction—estimating the labels of the specific test points one cares about, rather than an intermediate function defined over the whole input space—on the principle that one should not solve a harder problem than the one actually posed. Full conformal prediction (Section 6) is transduction made exact and distribution-free, and the predictive-Bayes object is its probabilistic completion: both decline to treat the parameter as the goal and aim straight at the next observation. The dispute between the cultures, then, was never really about stochastic models versus algorithms; it was about whether the parameter or the prediction is primitive, and de Finetti settled that question in favor of prediction in 1937. That settlement is the subject of the next section, which supplies the theorem the rest of the paper leans on.

## 3. The Predictive Foundations of Bayes

The spine of the argument is the predictive view: the parameter is a derived construct, and the primitive is a coherent sequence of predictive distributions. For an infinite exchangeable sequence, de Finetti [13] showed the joint law factors as a mixture of i.i.d. laws,

(1) $$p\left(x_{1},\ldots ,x_{n}\right)=\int \left(\prod_{i=1}^{n}F\left(x_{i}\right)\right)\;d\pi \left(F\right),$$

so that the prior–likelihood factorization is *induced* by exchangeability rather than presupposed; the parameter *F* (or a finite-dimensional θ indexing it) appears only as the almost-sure limit of an empirical functional; the information a sample carries about that limit is itself a different quantity from the information it carries about the next observation, as Section 9 makes precise [14]. Diaconis and Freedman [15] sharpened this for finite and partially exchangeable sequences, while Doob [16] supplied the martingale consistency theorem by which a sequence of predictives recovers the posterior. Fortini et al. [17] characterized when a predictive sequence is generated by a parametric model through predictive sufficiency. Geisser [18] and the prequential principle of Dawid [12] then elevated prediction, not estimation, to the criterion by which procedures are judged—the direct ancestor of every coverage- and calibration-flavored guarantee in the modern literature. In this light, the prior is not an extra assumption bolted onto a likelihood but a modeling choice about predictions—the exchangeable law one commits to before seeing data—which is exactly the choice an amortized learner makes when it fixes a pretraining corpus or a simulation distribution.

The contemporary revival is the theory of martingale posteriors [19,20]. Its move is to locate statistical uncertainty in the missing observations $Y_{n+1:\infty }$ rather than in a prior on θ. One specifies a sequence of one-step predictives and updates them through a predictive recursion [21]; a convenient choice is the bivariate-copula update of Fong et al. [19],

(2) $$p_{n+1}\left(y\right)=p_{n}\left(y\right)\left\{1-\alpha_{n+1}+\alpha_{n+1}\;c_{\rho }\left(P_{n}\left(y\right),P_{n}\left(y_{n+1}\right)\right)\right\},$$

with $c_{\rho }$ a bivariate-copula density and $\alpha_{n}$ a step size, which “predictively resamples” the future and reads off any functional θ=θ(F) of the completed population as the limit $\theta =\lim_{N\to \infty }\theta \left({\hat{F}}_{N}\right)$. Doob’s theorem guarantees that, in the exchangeable case, the law of θ so obtained is the Bayesian posterior. The construction extends to conditionally identically distributed (c.i.d.) sequences [22], which relax exchangeability while preserving the martingale that keeps the scheme coherent; recent asymptotics pin down when a predictive sequence collapses to a finite-dimensional likelihood [23]. This is the engine of the survey: *every later paradigm is a way of specifying the predictive and letting the martingale do the rest.*

Two classical computational shadows of this view prefigure the “sample, don’t integrate” ethos of generative computation. The Bayesian bootstrap [24] and the weighted likelihood bootstrap [25] are martingale posteriors under the empirical distribution predictive, and the Pólya-urn-to-Dirichlet-process equivalence [26] is the cleanest worked example of a predictive update that induces an exchangeable future. The foundations thus tell us *what* to compute; the next section is about *how*, and about the one design decision—amortization—that distinguishes the modern answer from the classical one.

## 4. From Inference to Computation: The Generative Turn and GBC

The second pillar is computational. The likelihood-free tradition—Approximate Bayesian Computation (ABC)—already reframed inference as simulation: ABC is essentially a nearest-neighbor smoother that tilts a simulated training set $\left\{\left(\theta^{\left(i\right)},y^{\left(i\right)}\right)\right\}$ toward the observed *y*, with accept–reject sampling as its weakness in high dimensions. Its modern descendant is *simulation-based inference* [6]: neural posterior, likelihood, or ratio estimation powered by normalizing flows [27,28] and by classification or adversarial surrogates [29]. Neural Bayes estimators [30] make the bargain explicit—pay a large upfront simulation cost and obtain near-instant inference thereafter.

Amortized variational inference is the deep learning-native version of the same bargain [31]. The encoder of a variational autoencoder [32] is a network trained to emit the approximate posterior for any input, so inference is once again a forward pass rather than a per-datum optimization [8]. Whether the amortized target is a posterior (simulation-based inference, VAE encoders), a parameter estimate (neural Bayes estimators), or a predictive density (the next section), the move is identical: replace per-instance computation with a single map learned over a distribution of instances.

*Generative Bayesian Computation* (GBC) is the sharpest statement of this turn [5]. Rather than approximate a density, GBC learns a transport map that pushes a base variable τ (e.g., uniform or Gaussian) to a posterior draw,

(3) $$\theta =Q\left(\tau ,y\right),\;\tau ∼U{\left(0,1\right)}^{d},\;\theta ∼p\left(\theta |y\right),$$

estimated as ordinary supervised learning on a simulation table by minimizing a check loss so that *Q* is the conditional quantile function,

(4) $$\hat{Q}=\arg\min_{Q}\;\frac{1}{N}\sum_{i=1}^{N}\rho_{\tau^{\left(i\right)}}\;\left(\theta^{\left(i\right)}-Q\left(\tau^{\left(i\right)},y^{\left(i\right)}\right)\right),\;\rho_{\tau }\left(u\right)=u\left(\tau -1\{u<0\}\right).$$

The method is thus *density-free*: it never forms a likelihood, applies wherever the data-generating process is given by deterministic latent updates or moment constraints, and reduces inference to a forward pass. The program now spans maximum expected utility, where the optimal action solves

(5) $$a^{★}\left(y\right)=\arg\max_{a}E\left[U(a,\theta )|y\right]=\arg\max_{a}\int_{0}^{1}U\left(a,Q(\tau ,y)\right)\;d\tau ,$$

so that expected utility is computed as a *marginal of quantiles*, illustrated with a fractional-Kelly portfolio [33]; and causal inference, where density-free quantile networks handle econometric data-generating processes and represent treatment effects as transport [34]. Ongoing work extends GBC to sequential state-space models, Schrödinger-bridge and survival variants, and probabilistic graphical models.

In short, simulation-based inference, neural Bayes estimators, prior-data fitted networks (Section 7), and GBC are *the same idea*—amortized posterior or posterior-predictive maps learned by simulation—differing only in target (parameter versus predictive) and parameterization (flow versus quantile network versus transformer). GBC’s distinctive contribution is the density-free quantile route, which also describes diffusion run backward (Section 7.3).

**Figure 3:**
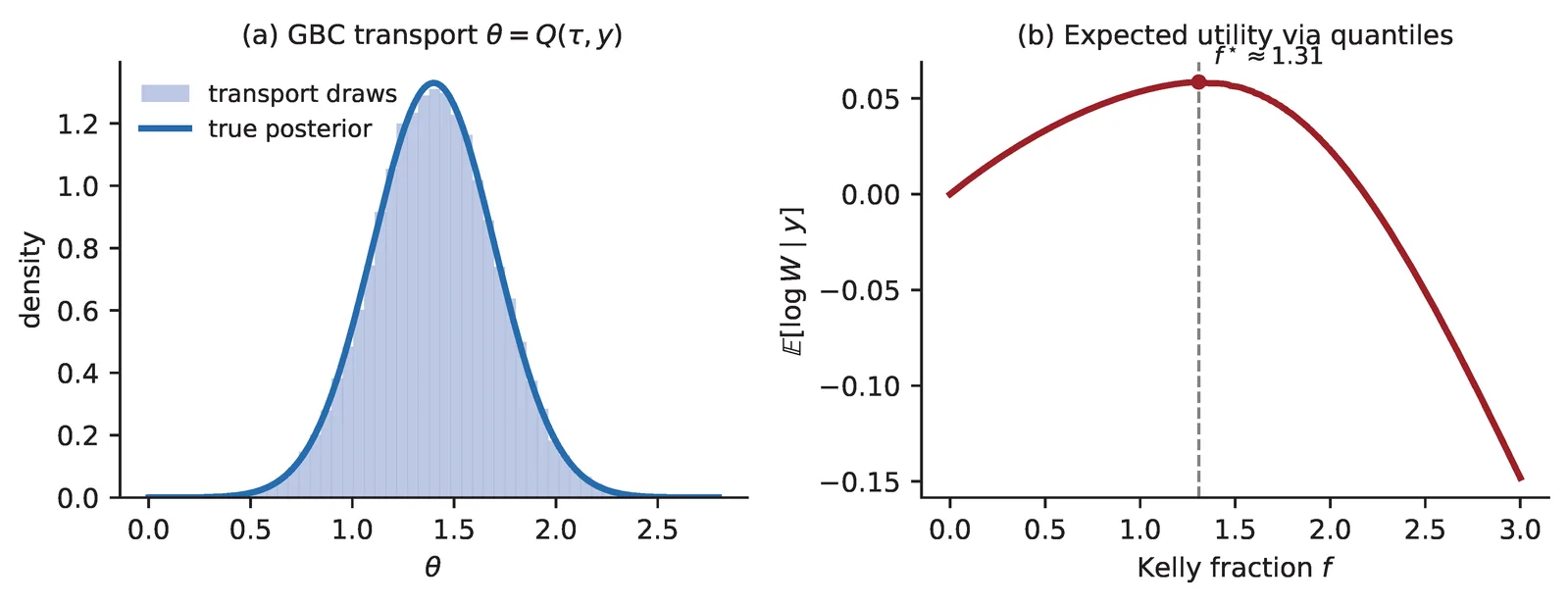
Generative Bayesian computation in miniature. (**a**) For a conjugate Gaussian location model, the transport θ=Q(τ,y) with τ∼U(0,1) reproduces the exact posterior; in non-conjugate problems, the same map is learned from a simulation table. (**b**) Expected utility as a marginal of quantiles: the expected log-wealth of a Kelly portfolio, $\int_{0}^{1}\log\;\left(1+f\;Q_{R}\left(\tau \right)\right)\;d\tau$, is maximized at the optimal fraction $f^{★}$ without ever forming a posterior density.

Figure 3 makes this concrete. In the conjugate Gaussian case, the learned map coincides with the analytic posterior quantile function, so transport sampling is exact; in a simulator-defined model, the same architecture is trained on the table and no analytic form exists. Expected utility (Figure 3b) then needs no separate integration—it is a one-dimensional average of the utility composed with the quantile map, the decision-theoretic counterpart of the alignment objective of Section 7.1.

### 4.1. Amortized, Sequential, and Per-Instance Inference

“Amortized” is easily confused with “online,” and the two are orthogonal. *Per-instance* inference solves a fresh optimization or simulation problem for each dataset: MCMC, per-datum variational inference, full conformal refitting. *Amortized* inference pays a one-time cost to learn a map over a *distribution of datasets* drawn from a simulator or a corpus, and thereafter answers any new instance by a forward pass; simulation-based inference, VAE encoders, neural Bayes estimators, prior-data fitted networks, and GBC are all of this type, and all accept an *amortization gap*—the excess loss of a single shared map relative to instance-specific inference [35]—in exchange for near-zero marginal cost. *Sequential* (online) inference updates as data arrive in time: particle filters and sequential Monte Carlo [36], the predictive recursion of Equation (2), adaptive conformal inference [37], and the anytime-valid e-values of Section 6. In short, amortization is offline and horizontal (across problems); sequential inference is online and vertical (within a problem).

The two compose rather than compete. An amortized transport can be dropped inside a particle filter, so that each sequential update is a forward pass rather than an inner optimization; conversely, a sequential scheme supplies the training targets on which an amortized map is fit. GBC for state-space models is exactly this composition—$\theta_{t}=Q\left(\tau ,y_{1:t}\right)$ trained offline on simulated trajectories and evaluated online—and a transformer doing in-context learning is the extreme case, in which the sequential update *is* the forward pass.

### 4.2. Why GBC as the Organizing Spine?

Three reasons, none of them a claim of empirical superiority. First, *minimality of assumptions*: GBC requires only the ability to *simulate*—no likelihood, no density, not even a differentiable one—which is the weakest interface any of these methods ask for, and the interface a modern simulator or structural econometric model actually offers; a framework that organizes the field should sit at the lowest common denominator. Second, *the quantile representation is the common form*: Equation (3) writes inference as a map from a base variable to a target draw, and normalizing flows, flow matching, and diffusion differ only in how that map is parameterized and trained (Section 8); writing it as a conditional quantile function makes the shared structure visible and makes expected utility the one-dimensional integral (5). Third, *it closes the loop to decisions*: that identity and the KL-regularized policy of Equation (12) are the same computation, which is what carries the framework into stochastic control and logistics (Section 10.2). Martingale posteriors supply the *foundations*, transport supplies the *mechanism*, and GBC supplies the *computational spine* that touches both.

## 5. Deep Learning as Bayesian Inference

Reading through the Bayesian lens of Polson and Sokolov [38], the core dualities of deep learning are immediate. Penalized training is maximum a posteriori estimation,

(6) $$\hat{w}=\arg\min_{w}\left\{\sum_{i}\ell \left(y_{i},f_{w}\left(x_{i}\right)\right)+\lambda \;r\left(w\right)\right\}=\arg\max_{w}\left\{\log p(y|w)+\log\pi (w)\right\},$$

so that regularizers are log-priors: the ridge corresponds to a Gaussian prior, the lasso to a Laplace prior, and the horseshoe to a global–local scale-mixture prior that behaves like an adaptive, signal-preserving regularizer [39]. Dropout is an approximate variational posterior [40], and the stationary distribution of stochastic gradient descent is an implicit posterior. The infinite-width limit makes the reading rigorous: wide networks converge to Gaussian processes [41], the training dynamics are governed by the neural tangent kernel [42], and marginalization—not point estimation—is what reconciles interpolation with generalization [43]. Approximation-theoretic guarantees for deep ReLU networks [44] underwrite the function classes on which GBC and prior-data fitted networks rely. On this account, the double-descent phenomenon [45] is a feature rather than a bug: averaging over a rich hypothesis class is exactly what a Bayesian does, so interpolation need not imply overfitting.

The connection runs in both directions. Deep ensembles [46], the most reliable uncertainty method in practice, are an approximate Bayesian model average over modes of the loss surface [43]; conversely, the cold-posterior effect—the empirical observation that down-weighting the likelihood or sharpening the posterior often improves held-out accuracy [47]—signals that the implicit prior and the data-augmentation-modified likelihood of a trained network differ from the nominal Bayesian ones, a discrepancy best read through the generalized-posterior lens of Section 9. The correspondences between common regularizers and their prior counterparts are collected in Table 1.

The dynamics, not just the optima, are Bayesian. Stochastic gradient Langevin dynamics turns the optimizer itself into a posterior sampler by injecting calibrated noise into each step [48], and the stationary distribution of plain stochastic gradient descent with constant step size approximates a Gibbs measure whose temperature is set by the learning-rate-to-batch-size ratio—the same Gibbs object as Section 9. Generalization theory has caught up with intuition: benign overfitting shows that interpolating estimators can still be consistent when the spectrum of the feature covariance spreads risk across many low-variance directions [49], a finite-sample mechanism for the marginalization story of Wilson and Izmailov [43] and for double descent.

### 5.1. A Penalty Is a Prior; A MAP Is Not a Posterior

Equation (6) is an identity between two *optimization problems*, and it should not be made to do more work than it can: the minimizer of a penalized loss is the *mode* of a posterior, not the posterior. Three gaps separate them. *The mode is not the measure*: in high dimensions, the mode can be unrepresentative of posterior mass—the lasso’s MAP is exactly sparse while the Laplace posterior puts zero mass on sparsity—so shrinkage behavior and uncertainty quantification can disagree, and nothing in (6) delivers a credible interval, a posterior variance, or a coverage statement. *Uncertainty must be added, not read off*: recovering the measure requires marginalization—sampling [48], ensembling [46], variational approximation [8], the infinite-width limit [41], or the amortized transports of Section 4—and each is a distinct estimator with a distinct error, about which the regularizer–prior dictionary is silent. *The implicit prior is not the nominal one*: what a trained network carries is a composite of penalty, architecture, initialization scale, optimizer bias, stopping time, and data augmentation, and the cold-posterior effect [47] is the cleanest evidence that the composite differs from the nominal Bayesian object—a discrepancy to be measured, not explained away.

We therefore use “regularizer = prior” as a *dictionary of correspondences between objectives* and reserve “Bayesian inference” for procedures that produce a distribution. In the taxonomy of Section 10.1, the MAP identity is graded E (exact, but about modes), the infinite-width posterior A, and the posterior of a finite trained network ≈.

### 5.2. The Horseshoe Beyond Shrinkage: Selection, Inference, and Robustness

Our use of the horseshoe [39] above is deliberately narrow—it is the prior whose shrinkage profile explains what an adaptive regularizer does—but that understates the role the family now plays in *inference*. On *selection*, the horseshoe+ prior [50] sharpens the tails and improves separation of signal from noise, and the regularized horseshoe [51] controls the slab so that large coefficients are not left entirely unshrunk—a practical necessity in logistic and weakly identified models. On *uncertainty quantification*, van der Pas et al. [52] show that horseshoe credible intervals attain honest coverage for signal coefficients under sparsity, with the familiar caveat near the detection boundary; this matters for Section 5.1, since the horseshoe is one of the few shrinkage priors for which the *posterior*, and not merely the mode, has been vindicated. On *robustness*, the family—horseshoe, horseshoe+, regularized horseshoe—has recently been examined under robust likelihoods in high dimensions, with efficient Gibbs samplers and the finding that one-group priors deliver valid credible intervals under heavy-tailed errors even without exact sparsity [53].

This is not a detour from the thesis. A global–local prior is a *scale mixture*, and a scale mixture is a transport (Section 8): $\beta_{j}=\lambda_{j}\tau z_{j}$ with $z_{j}∼N\left(0,1\right)$ is precisely a map from a base variable to a target draw, which is why the horseshoe is the natural prior to *simulate from* inside a GBC training table and why robust (check loss, Huber, quantile) likelihoods pose it no difficulty. The selection and coverage literature is what tells us this transport can be trusted for inference and not only for prediction.

### 5.3. Tree Ensembles: The Dictionary Beyond Smooth Models

Breiman’s own exhibit for the algorithmic culture was a random forest [54], not a neural network, and gradient boosting [55] still leads on tabular data, so it is fair to ask where tree ensembles sit in a dictionary stated for smooth models. They fit it through a different door: their regularization is *structural* rather than additive, and their Bayesian counterpart is a prior over *functions*—tree topologies and leaf values—rather than over weights. Three correspondences, recorded in the lower block of Table 1, do the work. *Averaging is marginalization*: bootstrap resampling with random feature subsets induces a sampling distribution over a function class, and the ensemble average is a Monte Carlo estimate of a predictive mean under it—the model-averaging reading Wilson and Izmailov [43] give for deep ensembles. *Shrinkage is explicit*: the boosting learning rate ν is a shrinkage prior on leaf values; depth limits, minimum leaf size and column subsampling are a complexity prior on topology; and small-ν stagewise fitting behaves like the early stopping that Table 1 already lists as an implicit Gaussian prior. *BART makes the prior explicit*: Bayesian additive regression trees [56] place a regularization prior on depth and leaf magnitudes that keeps each tree a weak learner, then sample the posterior over the sum-of-trees function, delivering credible bands rather than a point predictor—with posterior concentration rates [57] and sparsity-inducing splitting priors [58] supplying the theory.

Trees therefore occupy the same row of the Rosetta table as deep networks: they represent p(y∣x), are trained by a proper score, keep their uncertainty in the ensemble, and hide their prior in the randomization and the complexity controls. The one real difference is smoothness, and it cuts in the trees’ favor exactly where the smooth prior is wrong. The amortization thesis has meanwhile reached this territory from the other side: prior-data fitted networks [59] beat tuned boosting on small tabular problems by *learning the posterior predictive of a prior over tabular data-generating processes*—an amortized Bayesian answer to the last stronghold of the algorithmic culture.

## 6. Conformal Prediction as Predictive Bayes

Split conformal prediction [7,60,61] fixes a nonconformity score s(x,y) (for example, the absolute residual of any fitted model), evaluates it on a held-out calibration set to obtain $s_{1},\ldots ,s_{n}$, and forms the prediction set from the empirical quantile of those scores,

(7) $$C_{\alpha }\left(x\right)=\left\{y:s\left(x,y\right)\leq {\hat{q}}_{1-\alpha }\right\},\;{\hat{q}}_{1-\alpha }=\left\lceil \left(n+1\right)\left(1-\alpha \right)\right\rceil \text{-}\mathrm{th}\;\mathrm{smallest}\;\mathrm{of}\;s_{1},\ldots ,s_{n}.$$

**Figure 4:**
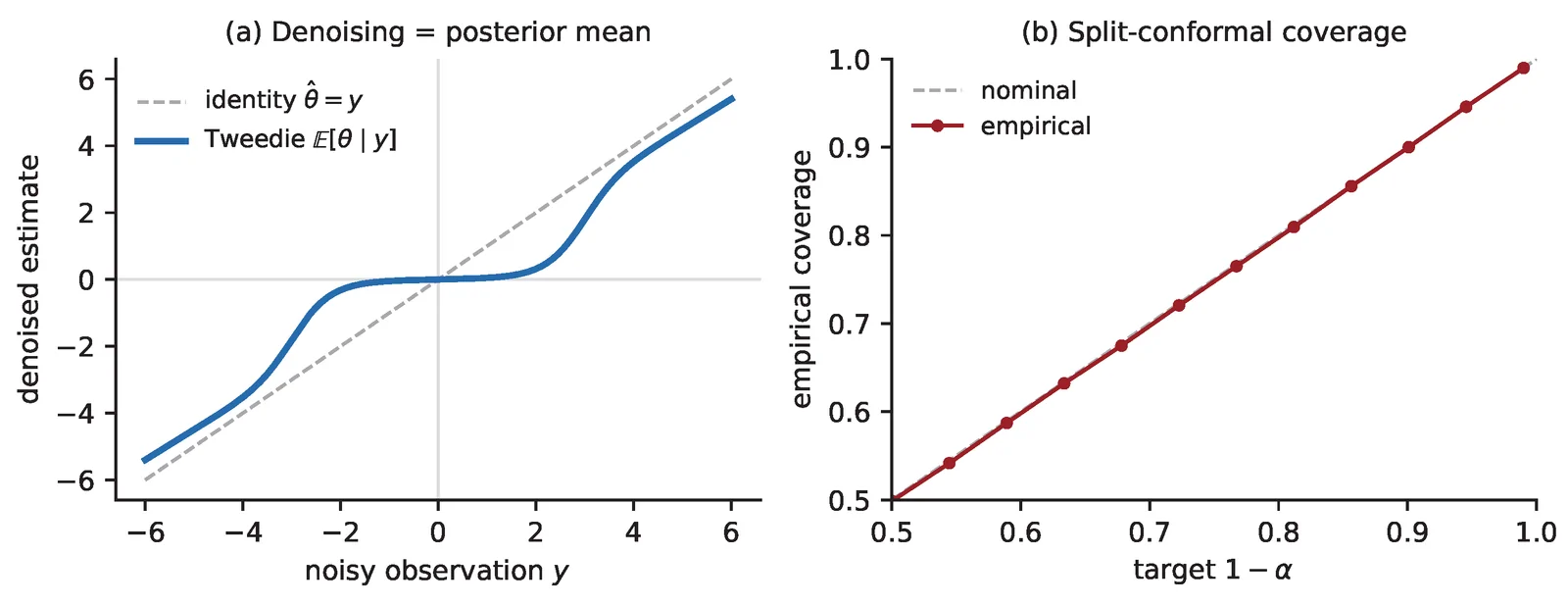
Two faces of the predictive calculus. (**a**) Tweedie’s formula turns denoising into an empirical-Bayes posterior mean: under a sparse (spike-and-slab) prior, the optimal estimate shrinks small observations toward zero and tracks the identity for large signals. (**b**) Split-conformal prediction attains its nominal coverage as a distribution-free consequence of exchangeability, the same symmetry underlying de Finetti’s theorem.

Exchangeability of calibration and test points then yields finite-sample marginal coverage,

(8) $$P\left(Y_{n+1}\in C_{\alpha }\left(X_{n+1}\right)\right)\geq 1-\alpha ,$$

with no distributional assumptions—the same symmetry that powers de Finetti is all conformal needs (Figure 4b). The bridges to Bayes are now well developed. Conformal Bayesian computation uses the posterior predictive as the nonconformity score, inheriting calibration and Bayes efficiency [62]. Bayes-optimal conformal sets, built from a correctly specified model, attain minimum expected volume at the target coverage [63]—the precise sense in which “Bayes is the efficient conformal.” The robustness extensions to covariate shift [64] and beyond exchangeability [65] map directly onto the c.i.d./martingale relaxations of Section 3.

A counterweight is in order. Recent analysis argues that conformal is better read as a rank-calibrated descendant of the Fisher–Dempster–Hill fiducial/direct-probability tradition than as Bayes proper: canonical conformal updates can depend on the marginal design P(X), can fail conglomerability, and need not be regular conditionals of any σ-additive exchangeable law. The defensible claim is therefore sharper than “conformal = Bayes”: conformal is the *frequentist-validity shadow* of the predictive-Bayes object, agreeing with it on marginal coverage but parting company on coherence under conditioning. In the grading of Section 10.1, this is the one row where the honest entry is not a grade of approximation but a qualified negative: the coverage guarantee is Exact and finite-sample, and the identification with Bayes fails under conditioning.

The base construction gives constant-width sets; the methods that made conformal practical restore input-dependence while keeping the exchangeability guarantee. Full (transductive) conformal refits the model with each candidate label appended and so uses the data most efficiently, but at a per-query computational cost; split conformal trades a one-time data split for near-free prediction, the same amortization bargain that distinguishes a trained map from per-instance inference elsewhere in this survey (Section 4.1). Conformalized quantile regression [66] takes the nonconformity score to be the signed distance to an estimated conditional quantile band, so the calibrated set widens and narrows with the heteroscedasticity of Y∣X—precisely the behavior a Bayesian posterior-predictive interval would exhibit, now distribution-free. Adaptive conformal inference [37] closes the loop online, updating the effective miscoverage level from realized errors so that coverage is maintained even under distribution shift, which is the empirical analogue of the c.i.d. relaxation of exchangeability in Section 3. The comparison with Bayesian credible sets is then clean: a well-specified Bayesian posterior-predictive interval is conditionally valid and efficient but loses coverage under misspecification; a conformal interval is marginally valid by construction but conditionally conservative. Using the Bayesian predictive as the conformal score [62,63] buys both—the efficiency of Bayes when the model is right and the coverage of conformal when it is not.

The same exchangeability that underwrites coverage also has a betting face. Game-theoretic probability replaces the coverage statement with a wealth process: testing by betting [67] builds an explicit nonnegative martingale that grows only when calibration fails, and the resulting e-values [68] are exactly the predictive likelihood ratios of Section 3, combinable by multiplication and valid under optional stopping. This gives conformal a sequential, anytime-valid form and connects it back to the martingale-posterior thread: a coherent predictor is one against which no betting strategy can accumulate wealth, and the de Finetti coherence condition is restated as a no-arbitrage principle. Conformal then calibrates the predictive object without modeling it. The next two sections turn to the methods that model it directly—first by learning the sequence of conditionals (language models), then by learning the transport that samples them (diffusion).

## 7. Language Models, In-Context Learning, and Generative AI

Autoregressive language modeling is sequential predictive inference. The chain-rule factorization

(9) $$p\left(x_{1:T}\right)=\prod_{t=1}^{T}p\left(x_{t}|x_{<t}\right)$$

is a sequence of one-step predictives, and next-token training under log-loss is proper-scoring-rule estimation of those predictives—a learned, amortized version of the de Finetti construction of Section 3. The theoretical literature makes the Bayesian reading explicit. If the pretraining corpus is a mixture over latent “concepts” *z*, then conditioning on a prompt performs posterior inference over the concept in the forward pass,

(10) $$p\left(x_{t+1}|x_{1:t}\right)=\int p\left(x_{t+1}|z\right)\;p\left(z|x_{1:t}\right)\;dz,$$

which is the mechanism by which in-context learning emerges [69]; high-capacity transformers empirically track the Bayes-optimal predictor across function classes [70]. The phenomenon is also visible mechanistically—few-shot ability appears abruptly at scale [71] and is carried in part by “induction heads” implementing copy-and-complete circuits [72]—and the mechanistic and Bayesian readings are complementary, since the circuit is *how* the forward pass approximates the posterior predictive. The crucial caveat comes from a martingale stress test: a genuinely Bayesian predictor must satisfy a martingale property and exchangeability, and real language models only partially pass these tests [73]; predictions are, for instance, sensitive to the order of in-context examples, a direct violation of the exchangeability a de Finetti predictor must respect. The equivalence is thus *aspirational and measurable* rather than exact—grade ≈ in the taxonomy of Section 10.1.

The missing link is the prior-data fitted network: a transformer trained on synthetic datasets drawn from a prior to output the posterior predictive directly, by minimizing the expected predictive negative log-likelihood over prior draws,

(11) $$\min_{\phi }\;E_{p(D,x,y)}\left[-\log q_{\phi }\left(y|x,D\right)\right],$$

whose minimizer is exactly the Bayesian posterior predictive under that prior [59,74]. Prior-data fitted networks are therefore GBC for the predictive, a transformer in place of a quantile network; the pairing is the predictive-amortization thesis at its sharpest. Neural processes [75] are the meta-learning sibling—amortized, exchangeability-respecting predictive maps. Note the grading discipline here: the minimizer of (11) *is* the posterior predictive as a population identity (E), while a network trained to finite precision on finitely many prior draws attains it only approximately (≈). The identity is a statement about the objective, not about the artifact.

### 7.1. Alignment as Bayesian Decision Theory

Pretraining estimates the predictive; alignment chooses an action under it. Reinforcement learning from human (or verifiable) feedback [76,77] fits a reward $r_{\psi }$ to preference data and then tunes the policy $\pi_{\phi }$ to maximize the expected reward under a Kullback–Leibler leash to the pretrained model $\pi_{0}$,

(12) $$\max_{\phi }\;E_{x∼\pi_{\phi }}\left[r_{\psi }\left(x\right)\right]-\beta \;\mathrm{KL}\left(\pi_{\phi }\;∥\;\pi_{0}\right),$$

whose solution is the Gibbs-tilted policy $\pi_{\phi }\left(x\right)\propto \pi_{0}\left(x\right)\exp\left\{r_{\psi }\left(x\right)/\beta \right\}$. This is exactly maximum expected utility against a predictive prior, the same object GBC computes as a marginal of quantiles in Equation (5): the reward is the utility, $\pi_{0}$ is the prior predictive, and β sets the relative-entropy budget. Evaluation is likewise predictive and information-theoretic: perplexity is the exponentiated predictive cross-entropy, $\mathrm{PPL}=\exp\{-\frac{1}{T}\sum_{t}\log p\left(x_{t}|x_{<t}\right)\}$, so minimizing log-loss is minimizing predictive entropy, tying the training objective to Section 9.

### 7.2. β Is a Temperature: Entropy Regularization and KL Control

Equation (12) is not merely *like* the objectives of maximum-entropy reinforcement learning; it is a one-step instance of them, and the coefficient doing the work is an inverse temperature. Write the KL leash against a *uniform* reference $\pi_{0}$ and (12) becomes the entropy-regularized objective $\max_{\pi }E_{\pi }\left[r\right]+\beta \;H\left(\pi \right)$, solved by the Boltzmann policy π(x)∝exp{r(x)/β} with optimal value of the free energy $\beta \log\sum_{x}\exp\left\{r\left(x\right)/\beta \right\}$—a log-sum-exp, or “soft max,” rather than a hard max. Extended over trajectories, this is maximum-entropy RL—maximum-entropy inverse RL [78], soft Q-learning [79], soft actor–critic [80]—which replaces the Bellman backup with its soft counterpart,

(13) $$V\left(s\right)=\beta \log\int \exp\left\{\frac{1}{\beta }\left(r\left(s,a\right)+\gamma \;E\;V\left(s^{\prime }\right)\right)\right\}\;da,\;\pi^{★}\left(a|s\right)\propto \exp\left\{\frac{1}{\beta }\;Q\left(s,a\right)\right\},$$

the general statement—control as inference—being that a KL-regularized control problem has a Gibbs-measure solution over trajectories [81]. In the linearly solvable MDPs of Todorov [82] and the path-integral control of Kappen [83], the KL cost is not an add-on but the *definition* of the control cost, which is why the Bellman equation linearizes under the exponential transform.

Three consequences matter for this survey. *The temperature is a prior weight*: large β keeps the policy close to the prior predictive and its entropy high (exploration), small β drives it to the reward-maximizing mode (exploitation), so the exploration–exploitation dial is the prior-strength dial—the same dial the Gibbs posterior of Equation (15) calls the learning rate and Bayesian tempering calls the temperature. *It is the same Gibbs object throughout*: the tilted policy $\pi_{0}\exp\left\{r/\beta \right\}$, the Gibbs posterior, the SGD stationary distribution whose temperature is the learning-rate-to-batch-size ratio (Section 5), and the Schrödinger bridge’s entropic regularizer (Section 8) are one exponential tilt seen four times—and the cold-posterior effect [47] is then just the observation that the empirically best β is not 1. *Softness is what makes the transport well posed*: β→0 recovers a deterministic optimal-transport map, while β>0 gives the entropically regularized problem—the Schrödinger bridge—which is why diffusion samples rather than collapses.

### 7.3. Diffusion as Empirical-Bayes Denoising

Score-based diffusion closes the loop. For Gaussian corruption y=θ+σε, Tweedie’s formula [84,85] expresses the posterior mean exactly through the score of the marginal,

(14) $$E\left[\theta |y\right]=y+\sigma^{2}\nabla_{y}\log m\left(y\right),\;m\left(y\right)=\int p\left(y|\theta \right)\;\pi \left(\theta \right)\;d\theta ,$$

so the optimal denoiser is an empirical-Bayes posterior mean and the learned score is a minimum mean-squared error reconstruction (Figure 4a). Denoising score matching makes the estimation tractable [86], each reverse-diffusion step is a posterior-mean update, and conditional generation is Bayes’ rule applied to the score [87,88]. Posterior sampling with denoising oracle results give the inverse-problem reading [89], and extensions of Tweedie beyond Gaussian noise to geometric-Brownian, squared-Bessel, and Cox–Ingersoll–Ross processes connect diffusion to the Bessel-process and generalized-gamma-convolution machinery relevant to heavy-tailed and financial settings [90]. The one-sentence synthesis: *diffusion is GBC run backward*—both learn a transport from a base distribution to the target, diffusion as a denoising score, and GBC as a quantile map.

## 8. Generative Models as Transport

Behind the specific architectures lies a single abstraction: a generative model is a *measure transport* that pushes a simple base law ρ onto a target μ, and in every Bayesian instance, the target is a posterior or posterior-predictive. Normalizing flows [27,28] learn an invertible map with tractable Jacobian; continuous-time flows and flow matching [91] learn the velocity field of an ordinary differential equation that carries ρ to μ; diffusion [87,88] learns the drift of a reverse-time stochastic differential equation, which Tweedie identifies with a sequence of posterior means (Section 7.3); and GBC learns the conditional quantile map Q(τ,y) directly. These differ in parameterization, not in kind. They also trace a spectrum: flow matching trains a simulation-free deterministic transport by regressing a velocity field [91], diffusion trains a stochastic transport by denoising score matching [86], and GBC trains a conditional quantile transport by a check loss—trading off sampling cost, training stability, and the tractability of likelihoods and utilities.

The organizing principle is optimal transport [92]: among all maps carrying ρ to μ, the Monge–Kantorovich optimum is the gradient of a convex potential (Brenier’s theorem), and its entropically regularized version is the *Schrödinger bridge*—the law on paths closest in relative entropy to a reference Brownian motion subject to fixed endpoints [93]. The Schrödinger bridge is therefore the diffusion that interpolates the base and target with minimal entropy production, its drift the Föllmer drift, and its regularizer the same Kullback–Leibler functional that defines the Gibbs posterior of Section 9 and the temperature β of Section 7.2. The Bayesian reading is then uniform: generative AI learns a transport to a posterior target, and entropy is the cost functional that selects the bridge. This is the natural home for GBC’s diffusion and Schrödinger-bridge variants and for the heavy-tailed, Bessel-process generalizations of Tweedie noted in Section 7.3.

## 9. The Information-Theoretic Glue

What licenses calling all of these methods “Bayesian” is that they are fit, evaluated, and regularized by the same information-theoretic quantities—a landscape of entropies, divergences, and mutual information whose interrelations are surveyed by Ebrahimi et al. [94]. Four observations do the work. First, *proper scoring rules* [95]: every method above optimizes a proper score, and the log-score is predictive cross-entropy, −Elogq(Y), whose expectation decomposes into the irreducible entropy of *Y* plus the relative entropy KL(p∥q) of the predictor from the truth—so “predict well” means “minimize predictive relative entropy,” and perplexity (Section 7.1) is just its exponential. Second, *generalized and Gibbs posteriors* [96,97]: the prior-to-posterior map is the solution of a relative-entropy-regularized risk minimization,

(15) $$q^{★}=\arg\min_{q}\left\{E_{\theta ∼q}\left[\sum_{i}\ell \left(\theta ;x_{i}\right)\right]+\mathrm{KL}\left(q\;∥\;\pi \right)\right\},$$

whose minimizer is the Gibbs posterior $q^{★}\left(\theta \right)\propto \pi \left(\theta \right)\exp\left\{-\sum_{i}\ell \left(\theta ;x_{i}\right)\right\}$, recovering Bayes’ rule when *ℓ* is the negative log-likelihood and the variational free energy (the ELBO) when *q* is restricted to a tractable family [8]. This is the objective machine learning optimizer descend; PAC-Bayes theory turns the same expression into a generalization bound [98,99], so Bayes is regularized empirical risk minimization in the Kullback–Leibler geometry, and the alignment objective of Equation (12) is its decision-theoretic twin. Third, *coding and large deviations*: minimum description of length identifies the two-part code length with the negative log-posterior, making MAP estimation optimal compression [100,101], while Sanov’s theorem makes the rate at which empirical evidence overwhelms the prior a relative entropy.

Fourth, and closest to this paper’s thesis, *information about the parameter is not information about the prediction*. Lindley’s measure of the information a sample carries about θ—the expected Kullback–Leibler divergence of the posterior from the prior—has a predictive counterpart, the corresponding measure for a future observation $Y_{n+1}$, and the two are distinct proper utilities. Ebrahimi et al. [14] study them jointly, showing how their relationship is governed by the prior, the sampling design, and the dependence in the sequence $Y_{i}|\theta$, and that the parameter measure dominates the predictive one under conditional independence while the ordering can reverse once that dependence is relaxed. This is the parameter/prediction dichotomy of Section 2 in information-theoretic form: an experiment that is highly informative about θ need not be the experiment one would run to predict well, so “how much have we learned?” has no answer until one says *learned about what*. It also sharpens the grading of Section 10.1: methods that amortize the predictive are optimizing the second measure, and their silence about the first is a design choice with a quantitative price.

Entropy is thus not a metaphor here but the common currency—of fitting (cross-entropy loss), of regularization (KL to the prior), and of evaluation (perplexity and calibration).

### 9.1. Bregman Divergences: The Class That Closes the Calculus

Relative entropy is not a coincidence but a member of a family, and naming the family turns a set of analogies into a calculus. For Φ, strictly convex and differentiable, the Bregman divergence [102] is the gap between Φ and its tangent plane,

(16) $$D_{\Phi }\left(p,q\right)=\Phi \left(p\right)-\Phi \left(q\right)-\langle \nabla \Phi \left(q\right),\;p-q\rangle ,$$

which gives a squared error when Φ is the squared norm, KL(p∥q) when Φ is negative Shannon entropy, and natural parameter divergence when Φ is an exponential family’s log-partition function. Four facts then organize everything above.

*Proper scoring rules are Bregman divergences.* By the Savage representation [95,103], every proper score satisfies $E_{p}S\left(q,Y\right)=-\Phi \left(p\right)+D_{\Phi }\left(p,q\right)$: a Bregman divergence from the truth, plus an irreducible term independent of the forecast. The log-score is the KL case, the Brier and continuous-ranked-probability scores the squared-error cases, and the reliability–resolution decomposition (Section 9.2) is the Bregman geometry of the identity. This is why “every method here optimizes a proper score” and “every method here minimizes a relative entropy” are the same sentence.

*Bayes’ rule and variational inference are Bregman projections.* Equation (15) is a KL projection of a tilted measure onto a family: exact onto all of P, and the ELBO onto a tractable subfamily—an *I*-projection in the sense of Csiszár, with the geometry of [104].

*EM is alternating Bregman projection.* The Csiszár–Tusnády reading [105] makes the E- and M-steps two projections in KL geometry, onto a data manifold and a model manifold—exactly the structure of the Blahut–Arimoto algorithm for channel capacity and rate distortion [106] and of iterative proportional fitting. Maximum-entropy computation, EM, and variational inference are one algorithm in three costumes.

*The check loss is not Bregman—and that is informative.* Quantiles are elicitable, but by a generalized piecewise-linear score rather than a Bregman one. This is the technical reason the density-free route of Equation (4) differs in kind: it targets a *functional* rather than a Bregman mean, which is why GBC needs no likelihood where a score-matching or maximum-likelihood method does.

The slogan: choose Φ, and you have simultaneously chosen a loss (fit), a regularizer (KL to the prior), a geometry, and an algorithm (alternating projection). Different corners of machine learning have chosen Φ differently; the choice, not the field, is what varies.

### 9.2. Calibration and Scoring in Practice

The predictive view comes with a ready-made evaluation protocol, and it is the one machine learning converged on independently. A predictive distribution is *calibrated* when its stated probabilities match empirical frequencies—probability-integral-transform values are uniform; reliability diagrams lie on the diagonal—and *sharp* when it is as concentrated as calibration allows. Proper scores such as the log-score and the continuous ranked probability score reward exactly this combination [95] and decompose into a reliability term and a resolution term —the Bregman decomposition of Section 9.1. Held-out log-loss, conformal coverage, and reliability curves are therefore not three unrelated diagnostics but three readings of one predictive object: log-loss is predictive cross-entropy, coverage is the calibration of an interval, and a reliability curve is the calibration of a probability. The prequential stance of Dawid [12] makes the protocol sequential—score each one-step-ahead forecast as the data arrive—which is precisely the regime in which the martingale tests of Section 7 and the e-values of Section 6 operate. Calibration without sharpness is vacuous (the climatological forecast is perfectly calibrated and useless), and sharpness without calibration is overconfidence; the predictive-Bayes object is the one that optimizes their proper-score trade-off, and every row of Table 2 is, in the end, judged by it.

## 10. Synthesis: A Unified Predictive Calculus

Table 2 assembles the equivalences developed above. Each row names a method, the predictive object it targets, the principle by which it is trained, where its uncertainty resides, where its prior is hiding, and—new in this version—*in what sense* the equivalence with Bayes holds; read together, the columns make the thesis concrete. The payoff for Breiman’s dichotomy (Section 2) is the calibration–entropy lens of Efron [2]: the algorithmic culture wins on prediction precisely when it performs amortized predictive Bayes and loses on attribution exactly where the predictive view is silent about θ.

### 10.1. Three Grades of Equivalence

Because the thesis is a claim of equivalence, its content depends on what “equivalent” means, and a survey that blurs the grades is doing rhetoric rather than mathematics. We fix three and use them consistently—in the abstract, in the text, and in the last column of Table 2.

E (*exact*): a mathematical identity at the population level, with no asymptotics and no estimation error. Tweedie’s formula (14) is an identity; the population minimizer of the PFN objective (11) *is* the posterior predictive; the population minimizer of the check loss (4) *is* the conditional quantile function; the Gibbs-tilted policy is exactly the solution of (12); split-conformal coverage is exact and finite-sample; the MAP identity (6) is exact *as a statement about modes*.

A (*asymptotic*): holds in a limit. De Finetti’s representation needs an infinite exchangeable sequence; Doob’s theorem delivers the posterior in the limit of predictive resampling; the network posterior is a Gaussian process in the infinite-width limit; the c.i.d. relaxation preserves the martingale but not exchangeability at finite *n*.

≈(*approximate, and measurably so*): holds up to estimation, optimization, and architectural error, and fails in identifiable ways. A trained language model is Bayesian only to the extent that it passes martingale and exchangeability stress tests, and it partially fails them [73]; a trained diffusion model’s score is an estimate; an amortized map carries an amortization gap [35]; a finite network’s implicit prior is not its nominal one [47].

The discipline is worth stating plainly. Where we write “= Bayes” with grade E, the claim is a theorem; with grade A, a limit theorem whose finite-sample behavior is a separate question; with grade ≈, an *empirical hypothesis with a designed test*—and the tests (martingale checks, exchangeability checks, calibration curves, coverage) are precisely the tools this framework hands the reader. Our thesis is not that trained artifacts *are* Bayesian; it is that the Bayesian predictive object is the target they approximate, that this identification tells us what to measure, and that the measured gaps are the interesting science.

### 10.2. Predictive Decision Theory in Operations: Logistics and Transportation

The framework’s natural habitat is any domain in which a decision must be made under uncertainty about a future observation, and logistics and transportation are the paradigm case. Three of our objects appear there directly. *The predictive object is the forecast*—traffic flow, arrival times, demand [107]—and what matters operationally is the distribution, not the point: a route that is fast in expectation and catastrophic in the tail is not a good route. *The decision is maximum expected utility as a marginal of quantiles*: with a learned quantile transport for travel time or demand, Equation (5) makes the cost of a plan a one-dimensional integral over τ, with no density and no inner MCMC loop—which is what makes it feasible inside an optimizer that must score many candidate plans, and which is the predictive-to-prescriptive program [108] with the predictive supplied by an amortized Bayesian map. *The KL-controlled objective is the exploration–exploitation trade-off in path planning*: in (12) and (13), β prices the willingness to try an unfamiliar route or probe an uncertain link against the expected cost of doing so—the setting for which linearly solvable MDPs [82] and path-integral control [83] were built. The transfer is not metaphorical: a logistics planner using an amortized predictive map, a KL-regularized policy, and a proper score for evaluation is running the same calculus as an aligned language model, with a travel-time distribution in place of a token distribution.

## 11. Discussion

To see the columns work together, trace a single row. Diffusion represents the predictive as a reverse-time score, is trained by denoising score matching, locates its uncertainty in the noise schedule and the data distribution, and hides its prior in that data distribution itself—with the equivalence exact at the level of the Tweedie identity and approximate at the level of the learned score. The conformal row answers the same questions with a prediction set, a rank-calibration objective, the empirical distribution of nonconformity scores, a frequentist shadow of the prior, and an exact coverage guarantee that is nonetheless *not* an identification with Bayes; the in-context-learning row, with a sequence of conditionals, next-token log-loss, a latent-concept posterior, the pretraining mixture, and an equivalence asymptotic in the ideal and approximate in the artifact. The Rosetta table is therefore not a list of loose analogies but one function—the predictive object—rendered in eight notations, each fixing the same four coordinates.

We have read conformal prediction, deep learning, tree ensembles, language models, and diffusion as a single predictive calculus: each constructs, calibrates, or samples the one-step-ahead density $p(y_{n+1}|y_{1:n})$, each is trained by a proper score, and each is amortized—computed once over a distribution of problems rather than re-derived per dataset. The unifying mechanism is measure transport to a Bayesian target (Section 8); the unifying currency is entropy, and more precisely, the Bregman class (Section 9); and the unifying foundation is de Finetti’s reduction in the parameter to a summary of an exchangeable predictive law (Section 3).

Earlier unifications anticipated parts of this picture: Wasserman [3,4] stressed the shared language of statistics and machine learning, and the Bayesian reading of deep learning [38] supplied the regularizer–prior dictionary. What the present synthesis adds is the organizing role of amortization, the identification of measure transport (Section 8) and relative entropy (Section 9) as the mechanism and the currency that make the equivalence operational rather than analogical, and the grading discipline of Section 10.1 that says, row by row, how much of the equivalence is theorem and how much is hypothesis.

### Where Prediction Is Not Enough

We have argued that prediction is the primitive object; we have not argued that it is the only one worth having. Four places deserve explicit mention because they are where a reader might over-apply the framework.

*Causal inference.* A predictive model estimates p(y∣x); an intervention requires p(y∣do(x)), and no predictive accuracy bridges the gap without assumptions that are not themselves predictive (ignorability, an instrument, a design). The classic pathology is a variable that is highly predictive *because* it is a consequence of the outcome: conditioning on it improves the score and destroys the causal reading. Amortization does not help and can hurt, since a map trained over a distribution of observational datasets will faithfully reproduce their confounding. GBC’s transports can *represent* treatment effects [34], but only once identification has been secured by an argument outside the predictive calculus.

*Parameter interpretation and identifiability.* De Finetti tells us the parameter is a summary of the predictive law; he does not tell us the summary is unique or interpretable. Two different mechanisms can induce the same exchangeable predictive, and a predictive-equivalent reparameterization leaves the forecast untouched and the science destroyed. Where the parameter is the scientific object—an elasticity, a rate constant, a treatment effect—the burden of identification falls where it always did.

*Explanation and mechanism.* Shmueli’s [9] separation is not dissolved by our argument, only relocated: a model selected to predict is generally not the model that explains, because the bias–variance trade-off that helps the first hurts the second. A predictive machine can be right for the wrong reason, and in science, the reason is the point.

*Hypothesis-driven discovery and extrapolation.* Prediction is validated on data exchangeable with the training distribution; hypotheses are interesting exactly when they license extrapolation to conditions not yet observed. Under shift, coverage weakens to the c.i.d./adaptive relaxations of Section 6, and an amortized prior trained on the old regime is the wrong prior for the new one. A mechanistic model that is worse on held-out data can be better on the intervention that has never been run.

The right summary is Efron’s [2]: prediction, estimation, attribution. Ours is a claim about the first, a claim about the computational form of the second, and—deliberately—no claim about the third. That is where Breiman’s two cultures genuinely diverge, and it is the subject of the first open problem below.

## 12. Open Problems

Five directions stand out. *(1) Coherence and calibration under shift.* The martingale tests of Falck et al. [73] and the beyond-exchangeability results of Barber et al. [65] suggest a common diagnostic for when amortized predictors stop being coherent; a unified theory is missing. *(2) What GBC still owes us.* Density-free quantile transport needs sharper guarantees on the approximation of multivariate conditional quantiles and on uncertainty propagation through the learned map. *(3) Attribution.* The predictive view is by design silent about θ; recovering an interpretable, identifiable structure from an amortized predictor—without surrendering its predictive accuracy—remains the central open problem bridging Breiman’s two cultures and the formal counterpart of Section Where Prediction Is Not Enough. *(4) Compute-optimal amortization.* When does paying the upfront simulation or pretraining cost beat per-dataset Markov chain Monte Carlo, how should that budget be allocated, and how large is the *amortization gap*—the suboptimality of a single learned map relative to instance-specific inference [35]? *(5) Priors at scale.* A pretraining corpus or simulation distribution is an implicit, high-dimensional prior; eliciting, auditing, and editing it is the form the prior-specification problem now takes, and it is far less understood than the low-dimensional parameter priors of classical Bayes.

## Figures and Tables

**Table 1:** Regularizers are log-priors. Each penalty r(w) added to the training loss corresponds to a prior $\pi \left(w\right)\propto e^{-\lambda r(w)}$, and the resulting estimator implements a characteristic shrinkage. The lower block extends the dictionary to tree ensembles (Section 5.3), where the “penalty” is a structural constraint on the function class rather than an additive term.

Regularizer	Implied Prior	Shrinkage
Ridge ($\ell_{2}$)	Gaussian	linear, uniform
Lasso ($\ell_{1}$)	Laplace	soft-threshold, sparse
Elastic net	Gaussian × Laplace	grouped, sparse
Group lasso	multivariate Laplace	block-sparse
Horseshoe	global–local scale mixture	adaptive, signal-preserving
Dropout	spike-and-slab (variational)	stochastic averaging
Early stopping	implicit Gaussian (shrink to init)	path-dependent
Boosting learning rate ν	shrinkage prior on leaf values	stagewise, ν↓ ⇒ stronger
Tree depth/min-leaf size	prior on tree topology (split-stopping)	complexity-penalizing
Number of trees × bagging	posterior over an ensemble of trees	variance reduction by averaging
BART’s regularization prior	explicit tree + leaf prior	each tree a weak learner

**Table 2:** A Rosetta table for the predictive calculus. Each modern method constructs, calibrates, or samples a predictive object; the source of uncertainty and the location of the implicit prior differ, but the predictive primitive is shared. The last column grades the equivalence: E = exact identity (in the population/idealized limit), A = asymptotic (holds in a limit: infinite exchangeable sequence, infinite width, n→∞), ≈= approximate for trained artifacts, with a measurable gap. See Section 10.1.

Method	Predictive Object	Trained by	Uncertainty in	Prior Hides in	Sense
Martingale posterior	$p(y_{n+1}|y_{1:n})$ sequence	predictive resampling/copula update	missing $Y_{n+1:\infty }$	predictive update	E (Doob); A for c.i.d.
GBC	transport τ↦θ∣y (quantile)	supervised learning on sim. table	base dist. + sim. model	sim. prior on θ	E at the population optimum; ≈trained
SBI/NBE/PFN	posterior or $p(y^{*}|D)$	amortized sim. training	base noise/sampled tasks	task-generating prior	E at the minimizer of (11); ≈trained
Deep nets (Bayes)	p(y∣x) w/marginalization	SGD + regularization	weight posterior/ensemble	regularizer = prior	E for MAP = mode; A infinite width; ≈else
Tree ensembles/BART	p(y∣x), sum-of-trees	greedy stagewise fit/MCMC	ensemble over trees	tree and leaf prior; randomization	E for BART; ≈for RF/GBM
Conformal	set $C_{\alpha }\left(x\right)$, coverage	rank calibration	empirical distribution of nonconformity scores (rank statistics)	frequentist shadow	E coverage; *not* Bayes under conditioning
LLM/ICL	$p(x_{t}|x_{<t})$	next-token log-loss	latent-concept posterior	pretraining mixture	A idealized; ≈real (fails martingale tests)
Diffusion	reverse-time score/denoiser	denoising score matching	noise schedule + data prior	data dist. as prior	E (Tweedie); ≈learned score

## Data Availability

No new data were created or analyzed in this study. Data sharing is not applicable to this article.
